# Editorial: Odyssey of Surfactant Proteins SP-A and SP-D: Innate Immune Surveillance Molecules

**DOI:** 10.3389/fimmu.2020.00394

**Published:** 2020-03-11

**Authors:** Uday Kishore, Roberta Bulla, Taruna Madan

**Affiliations:** ^1^Biosciences, College of Health and Life Sciences, Brunel University London, Uxbridge, United Kingdom; ^2^Department of Life Sciences, University of Trieste, Trieste, Italy; ^3^Department of Innate Immunity, ICMR—National Institute for Research in Reproductive Health, Mumbai, India

**Keywords:** SP-A, SP-D, inflammation, cancer, innate immunity, infection, polymorphisms

Surfactant protein A (SP-A) and D (SP-D) are hydrophilic collagenous C-type lectins, which were originally discovered in the lungs associated with surfactant phospholipids. It was later shown that the two proteins, unlike hydrophobic surfactant proteins, SP-B and SP-C, are keenly involved in protecting lungs against insults from pathogens, allergens, apoptotic, and necrotic cells (1). Two aspects became clear in subsequent years that (i). SP-A and SP-D have extra-pulmonary existence; and (ii). They can manipulate immune cells, and thus, regulate inflammatory responses (2). Although there have been a constant debate about their candidate receptor(s)—there are several reported so far (1). Much of the immunological studies, beyond interaction with surfactant system and pathogens, have been followed up toward SP-D. It has become apparent that SP-D is an innate immune surveillance molecule at the mucosal surfaces, which can act as a bridge between innate and adaptive immunity. The role of SP-D in modulating antigen presentation, helper T cell polarization and B cell differentiation and class switching (3) are few neat examples. This volume comprises 14 papers that extend our knowledge on SP-A and SP-D, and their roles in infection, inflammation and cancer.

A consistent theme discussed by several contributors is the differential role of two forms of SP-A in oxidative stress and lung innate immunity (Thorenoor, Umstead et al.; Nalian et al.; Thorenoor, Kawasawa et al.; Wang et al.). In humans, there are two SP-A variants differing in the collagen region, SP-A1 and SP-A2, encoded by SFTPA1 and SFTPA2, respectively, and produced by the alveolar type II cells in the lung. Importantly, SP-A1 and SP-A2 seem to differentially bind to phagocytic, but not to non-phagocytic cells (Thorenoor, Umstead et al.). SP-A1 and SP-A2 differentially bind and regulate neonatal and adult human alveolar macrophages (AMs) (Thorenoor, Umstead et al.). AMs from transgenic mice expressing human SP-A1 and SP-A2 exhibit differential expression of cell surface proteins (Thorenoor, Kawasawa et al.) Rodents express only one SP-A variant; thus, Nalian et al. have compared the rodent and human SP-A with respect to structural determinants of the function. The data infers that mouse SP-A is a functional hybrid of human SP-A1 and SP-A2. Particularly striking in this regard is the differential response in the two sexes. Humanized transgenic (hTG) male and female mice, carrying both SP-A1/SP-A2 (6A2/1A0, co-expressed) and SP-A-gene deficient mice were exposed to filtered air (FA) or ozone (O_3_), and miRNA levels were measured in isolated AMs (Thorenoor, Kawasawa et al.). The AM miRNome of co-expressed females was significantly downregulated in response to ozone induced oxidative stress. Several of the validated miRNA targets were involved in pro-inflammatory response, anti-apoptosis, cell cycle, cellular growth, and proliferation (Thorenoor, Kawasawa et al.). Continuing with this theme, Wang et al. have analyzed bronchoalveolar lavage (BAL) proteomic profile and associated signaling pathways in hTG SP-A1 and SP-A2 mice, as well as in SP-A knock-out mice exposed to O_3_ or *Klebsiella pneumoniae*. The hTG-SP-A2 mice showed significantly higher number of differentially expressed proteins, with the majority being increased in male mice while decreased in female mice. Survival of hTG mice (expressing SP-A1 alleles/ SP-A2 alleles/ Co-ex) challenged with *Klebsiella pneumoniae* was observed to be gene specific (co-ex and SP-A2 showing higher survival), variant-specific [co-expressed hTG (6A2/1A0) and hTG (1A0)] showing higher survival and sex specific (females showing higher survival). Cystic Fibrosis (CF) is characterized by altered SP levels. Lin et al. demonstrated that complex single nucleotide polymorphisms (SNPs)-SNP interactions of the surfactant genes may contribute to the pulmonary disease in CF patients. Wang Group have looked into SP-D gene polymorphisms and susceptibility to tuberculosis, by identifying/cloning two major SP-D exonic polymorphisms and examining their interaction with *Mycobacterium bovis* bacillus Calmette–Guérin (*M. bovis*) BCG. The authors seem to suggest that C92T (rs721917; amino acid rSP-D 92T variant) may increase susceptibility to TB (Hsieh et al.).

Hartshorn group have elegantly reviewed the molecular mechanisms used by SP-D to neutralize influenza A Virus (IAV) (Hsieh et al.). The group has been a pioneer in the field and the review summarizes the highlights of their credible work over a couple of decades. Al-Ahdal et al. have shown that a recombinant form of truncated human SP-D (rfhSP-D), composed of homotrimeric neck and C-type lectin domains, was able to restrict H1N1 and H3N2 subtypes of IAV (as well as their pseudotyped viral counterparts) from infecting A549 cells, a lung epithelial cell line, thus acting as an entry inhibitor. This is in contrast to the recombinant fragment of human SP-A that seems to promote IAV infectivity; however, full-length SP-A inhibits viral entry (4). These results seem to suggest that the two molecules i.e., SP-A and SP-D, especially their C-type lectin domains, can be very distinct in their functional properties; this point has been emphasized by other papers in this volume. One important point that needs mentioning is that rfhSP-D is also able to dampen the pro-inflammatory cytokine storm induced by IAV, which could minimize the lung injury caused by the virus. Next two papers are focused on how SP-D (rather rfhSP-D) can offer protection against HIV-1 at the mucosal surface. Dodagatta-Marri et al. show, for the first time, that DC-SIGN is a putative receptor for SP-D; the binding takes place via the C-type lectin domains of both proteins. This interaction is interestingly poised since both SP-D and DC-SIGN can interact with HIV-1. The authors show that opsonizing HIV-1 with rfhSP-D prior to viral exposure to DC-SIGN bearing cells reduced viral ability to get transferred to CD4^+^ T cells *in trans*. Thus, this is another layer of protection that rfhSP-D works at against HIV-1, and follows up earlier studies (5, 6). A seminal study by Pandit et al. in this volume reports use of vaginal explants and inhibition of HIV-1 transfer in an *ex vivo* context. The authors have also carried out a transcriptomics analysis revealing how rfhSP-D modulates a wide range of target cells and pathways in order to act as a mucosal barrier to HIV-1. In addition, the study has utilised a rabbit model of vaginal irritation in order to demonstrate that rfhSP-D is a safe prophylactic molecule for vaginal use.

Madan et al. showed nearly 20 years ago that rfhSP-D could offer therapeutic protection in a murine model of pulmonary hypersensitivity induced by *Aspergillus fumigatus* allergens/antigens (7). Subsequently, SP-D knock-out mice were found to be hyper-eosinophilic that could be ameliorated by rfhSP-D intranasal treatment (8). The mechanism of eosinophil clearance remained unclear until Mahajan et al. showed that eosinophils derived from allergic patients were susceptible to apoptosis induction by rfhSP-D (9) via p53 pathway, as revealed by proteomics analysis of a eosinophilic leukemic cell line, AML14.3D10 (10). This opened the area of SP-D mediated immune surveillance in cancer. It was subsequently shown that SP-D binds to EGF receptor on A549 cells and had an anti-proliferative and anti-invasive effect (11). In this volume, Kaur, Riaz, Murugaiah et al. reported the ability of rfhSP-D to induce apoptosis *via* TNF-α/Fas-mediated pathway regardless of the p53 status in human pancreatic adenocarcinoma (PDAC) using Panc-1 (p53^mt^), MiaPaCa-2 (p53^mt^), and Capan-2 (p53^wt^) cell lines. Treatment of these cell lines with rfhSP-D caused growth arrest in G1 cell cycle phase, triggered transcriptional upregulation of pro-apoptotic factors such as TNF-α, and induced apoptosis *via* Fas-mediated pathway in a p53-independent manner. In another paper, Kaur, Riaz, Singh et al. demonstrate that rfhSP-D is also capable of inhibiting TGF-β expression in the PDAC cell lines, and thus, suppressing their invasive-mesenchymal properties. The rfhSP-D-treated pancreatic cancer cell lines showed reduced expression in the cytoplasm of Smad2/3, suggesting that an interrupted signal transduction negatively affected the transcription of key mesenchymal genes. Thus, expressions of Vimentin, Zeb1, and Snail were found to be downregulated upon rfhSP-D treatment. Both these studies suggest that rfhSP-D can potentially be used to therapeutically target pancreatic cancer cells. A bioinformatics analysis using Oncomine dataset and the survival analysis platforms Kaplan–Meier plotter has been performed by Mangogna et al., which showed that in the lung, gastric, and breast cancers, there is a lower expression of SP-D than normal tissues. On the contrary, a higher expression than normal tissue was observed in ovarian cancer. In lung cancer, the presence of SP-D could be associated with a favorable prognosis. On the contrary, at non-pulmonary sites such as gastric, breast, and ovarian cancers, the presence of SP-D could be associated with unfavorable prognosis. All these data indicate that SP-D could also be used as a potential diagnostic marker.

In a critical assessment, Colmorten et al. have reviewed the role of SP-D in the vascular inflammation, inferring a dual role of SP-D in the development of atherosclerosis. A pro-atherogenic role of SP-D is evident from *in vivo* studies in SP-D knock-out mice. Clinical studies have shown a positive association between circulatory SP-D levels, carotid intima-media thickness, coronary artery calcification and risk of both total and cardiovascular disease mortality.

It is clear from the studies being reported in this volume that the field of SP-A and SP-D continues to grow and is now throwing a number of pleasant surprises. The therapeutic potential of rfhSP-D needs to be realized via planned clinical trials. This decade is going to be an exciting one for surfactant protein research.

## Author Contributions

All authors listed have made an equal, direct and intellectual contribution to the work, and approved it for publication.

### Conflict of Interest

The authors declare that the research was conducted in the absence of any commercial or financial relationships that could be construed as a potential conflict of interest.
